# P-169. Comprehensive Sexuality Education in Secondary Schools and Syphilis Incidence by Country: An Ecological Study

**DOI:** 10.1093/ofid/ofae631.374

**Published:** 2025-01-29

**Authors:** Michelle C Davidson, Kevin Ikuta

**Affiliations:** David Geffen School of Medicine at UCLA, Los Angeles, California; West Los Angeles VA, Los Angeles, California

## Abstract

**Background:**

Comprehensive sexuality education (CSE) is essential curriculum covering sexual and reproductive health topics, including transmission of sexually transmitted infections. CSE has been added as a Sustainable Development Goal (SDG) indicator (4.7.2) and is becoming more widespread, but the relationship between degree of CSE implementation and incidence of STIs has not been previously described. The aim of this study was to investigate whether countries with more widespread CSE in secondary schools have lower incidence rates of syphilis than those with limited CSE.

**Table 1:**
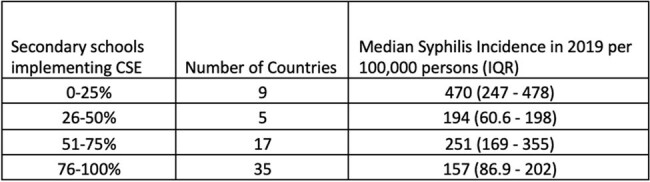
Percent of secondary schools implementing CSE in 2018 vs. median syphilis incidence in 2019.

**Methods:**

We used CSE implementation estimates from WHO as described in the 2021 UNESCO report: The Journey Towards Comprehensive Sexuality Education. This report utilizes data from multiple surveys to produce an estimate of the percent of secondary schools providing CSE by country in 2018, categorized as “0-25%”, “26-50%”, “51-75%”, or “76-100%”. We used syphilis incidence rates from the Global Burden of Disease Study 2019. We conducted a multivariable Poisson regression evaluating the relationship between percent of schools with CSE and syphilis incidence, adjusting for WHO region and income level.

**Figure:**
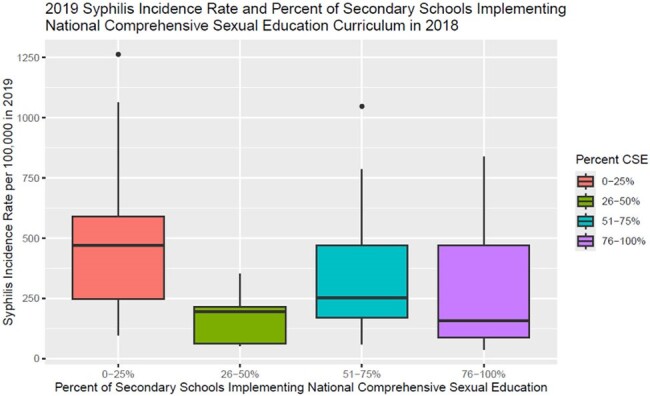

**Results:**

CSE data were available for 66 countries, all of which had syphilis incidence estimates available (Table 1). Countries that reported CSE in fewer than 25% of secondary schools had a syphilis incidence that was 1.83 (95% CI 1.78 – 1.89) fold higher than countries with CSE in greater than 25% of schools. This difference was attenuated but remained significant after adjusting for region and income level with a relative rate of 1.40 (CI 1.36 – 1.44). Each increase in CSE category was associated with a 26.6% (CI 25.9 – 27.4) relative reduction in syphilis incidence rate (Figure 1). This reduced to 7.1% (CI 6.2 – 8) when adjusted for region and income level.

**Conclusion:**

More widespread CSE is associated with lower syphilis incidence, likely by reducing unsafe sex practices, although a causal relationship cannot be inferred given study design. These findings suggest that countries with the greatest need for CSE are the ones with the most limited availability. Further research on this topic would be beneficial to more thoroughly evaluate how CSE programs impact rates of syphilis and other STIs.

**Disclosures:**

**All Authors**: No reported disclosures

